# Acacetin enhances the therapeutic efficacy of doxorubicin in non-small-cell lung carcinoma cells

**DOI:** 10.1371/journal.pone.0182870

**Published:** 2017-08-31

**Authors:** Reenu Punia, Komal Raina, Rajesh Agarwal, Rana P. Singh

**Affiliations:** 1 School of Life Sciences, Central University of Gujarat, Gandhinagar, Gujarat, India; 2 Cancer Biology Laboratory, School of Life Sciences, Jawaharlal Nehru University, New Delhi, India; 3 Department of Pharmaceutical Sciences, School of Pharmacy, University of Colorado Denver, Aurora, Colorado, United States of America; 4 University of Colorado Cancer Center, Aurora, Colorado, United States of America; University of South Alabama Mitchell Cancer Institute, UNITED STATES

## Abstract

**Background:**

Anthracyclines are efficient and potent agents to treat broad range of cancers but cytotoxicity induced by them limits their use in therapeutics. Use of plant-derived agents help to prevent or delay the process of cancer progression and their combination increases the anti-cancer potential of mainstream compound. However, multidrug resistance is major cause of treatment failure in cancer patients.

**Purpose:**

In this study, combination treatments of fisetin or acacetin with doxorubicin were explored for their potential synergistic effect on non-small-cell lung carcinoma (NSCLC) cells.

**Study design:**

During this study, NSCLC model cell lines A549 and H1299 were used to determine the combinatorial effect of phytochemicals namly acacetin and fisetin with doxorubicin.

**Methods:**

The effects of individual compounds and their combination on cell viability, clonogenic potential and cell cycle progression were studied. Efflux of doxorubicin was measured by spectrofluorophotometer, whereas accumulation inside the cells was analyzed by flow cytometry and confocal microscopy. Expression of MDR1 was checked by semi-quantitative PCR.

**Results:**

The results showed that the cell viability of A549 and H1299 cells were significantly decreased in time- and dose-dependent manner, although A549 cells showed more sensitivity toward doxorubicin than H1299 cells. Mostly, combination of doxorubicin showed good synergy with acacetin in both the cell lines whereas, fisetin exerted synergistic effect only at 72 h of treatment in H1299 cells. Acacetin with doxorubicin caused G2/M arrest by downregulating CDK-cyclin complex in A549 cells. Acacetin—doxorubicin combination decreased the clonogenic potential of A549 and H1299 cells upto 82% and 59%, respectively, as compared to control. Acacetin also decreased efflux of doxorubicin by 59% after 30 mins of exposure to A549 cells and further increased accumulation of doxorubicin inside the cells upto 55% in 2 h. The modulatory effect of acacetin-doxorubicin combination on doxorubicin influx and efflux was mediated through downregulation of MDR1 treansporter in NSCLC cells.

**Conclusion:**

These findings suggested that acacetin augments the cytotoxicity of doxorubicin at lower concentrations in lung cancer cells. Their combination leads to more retention of doxorubicin in the cells by modulating drug trasporter and thus enhances its therapeutic potential.

## Introduction

Lung cancer accounts for greater than 1.5 million new diagnosis per year, which represents 13% of total cancer diagnosis and caused 1.6 million of total cancer deaths worldwide in 2012. With very low 5-year survival rate, it has remained a life-threatening disease [1]. On the basis of histology, it is categorized into small-cell lung carcinoma (SCLC) and non-small-cell lung carcinoma (NSCLC). With 85% of all lung cancer cases, NSCLC is further classified into squamous cell carcinoma, adenocarcinoma and large cell carcinoma, which vary in their morphology and cell origin. Patients with advanced non-small-cell lung cancer survive only for 9–12 months [2]. Chemotherapy is an effective strategy to improve the quality of life and survival of cancer patients but some cancer patients do not respond to chemotherapy and become resistant to one or more therapeutic drugs. This leads to increase in the drug dosage, which in turn increase the cytotoxicity and undesirable effects to normal cells/tissues.

Multidrug resistance (MDR), ability of tumor cells to develop cross resistance towards structurally dissimilar drugs, remain a major limitation for the treatment of NSCLC patients with chemotherapeutic compounds [3]. Cells having MDR have overexpression of ATP binding cassette (ABC) transporters, which can attenuate the efficacy of drugs by pumping them actively outside the cells [4]. These transporters prevent the retention and cytotoxicity of drug inside the cells including anthracyclines, taxanes, vinca alkaloids, epipodophyllotoxins etc. [5].

Doxorubicin, an anthracycline antibiotic, is widely used and known for its anticancer activity towards lung, breast, ovarian, thyroid and gastric cancers [6]. The major limitation of doxorubicin use is cumulative toxicity leading to fatal congestive heart failure [7]. The response of doxorubicin towards pre-treated and treated patients varied between 28% and 43% in breast cancer patients [8]. Treatment of NSCLC cells with doxorubicin provided only 30–50% overall response [9]. Now a days, the major focus of doxorubicin research is to find an alternative approach to reduce its cytotoxicity and enhance its efficacy.

Flavonoids are part of our daily diet and well-studied for their pharmacological properties against many diseases including cancer. Acacetin (5,7-dihydroxy-4′-methoxyflavone), an O-methylated flavone is present in damiana (*Turnera diffusa*) and black locust (*Robinia pseudoacacia*). It is well known compound for its antiplasmodial, antimutagenic, antiperoxidant activities [10]. Fisetin (3,7,3′,4′-tetrahydroxyflavone), another commonly used flavonoid is present in many edible fruits and vegetables. Both phytochemicals exert antioxidant, anti-inflammatory and anti-proliferative activities towards many cancer cells [11,12]. These flavonoids are inexpensive, easily available and well-tolerated and may potentiate the cytotoxic effect of another agent [13]. Therefore, this study was undertaken to investigate whether fisetin or acacetin could act synergistically with doxorubicin and increase its cytotoxicity against lung cancer and to further explore the possible mechanism involved in the process.

## Materials and methods

### Cell lines and cell culture

The human NSCLC cell lines A549 and H1299 cells were purchased from ATCC (Manassas, VA). Both the cell lines were cultured in Roswell Park Memorial Institute (RPMI) 1640 medium (Gibco, Life Technologies, USA), supplemented with 10% (v/v) Fetal Bovine Serum (FBS; Gibco, Life Technologies, USA), 100 units/ml penicillin, 100 μg/ml streptomycin and 250 ng/ml amphotericin B at 37°C under 5% CO_2_ in humidified incubator (Thermo Fisher Scientific, Waltham, MA). The cells were cultured as adherent monolayer, media was changed every alternate day and passaged when cell confluency reached ~80%.

### Acacetin, fisetin and doxorubicin treatments

Fisetin, acacetin and doxorubicin were purchased from Sigma Aldrich, USA with molecular weight of 286.24 g/mol, 284.26 g/mol and 579.98 g/mol, respectively. For trypan blue dye exclusion assay 0, 5, 10, 25 and 50 μM concentrations of fisetin and acacetin were used and 10 and 25 μM fisetin and acacetin were selected for combination study. Both cell lines (A549 and H1299) were treated with 0, 10, 25, 50 and 100 nM doxorubicin and based on results, 10 nM doxorubicin for A549 and 25 nM doxorubicin for H1299 were selected for combination study, colony formation assay and cell cycle analysis. Fisetin and acacetin were used at 25 μM for all further experiments, whereas, 10 μM doxorubicin was selected for drug efflux and accumulation studies.

### Trypan blue dye exclusion assay

Cell viability of A549 and H1299 were analyzed with method described by Vundru et al [14]. After 72 h of treatment period, cells were collected and stained with trypan blue dye. Cells were counted and scored for total viable cells. DMSO was present in equal volume (0.1%, v/v) in all the treatment groups. Each experiment was repeated thrice.

### Cell cycle distribution analysis by flow cytometry

Cells were similarly seeded as cell viability assay and treated with selected concentrations of fisetin, acacetin, doxorubicin and their combinations. Cell were processed and resuspended in saponin/propidium iodide cocktail [0.3% saponin (w/v), 0.1 mM EDTA, 25 μg/ml PI (w/v) and 10 μg/ml RNAse (w/v)] in PBS in dark condition and incubated overnight at 4°C [15]. The cells were analyzed and data was acquired using fluorescence-activated cell sorter (FACS).

### Western immunoblotting

A549 and H1299 cells were treated with fisetin, acacetin, doxorubicin, fisetin-doxorubicin and acacetin-doxorubicin for 72 h to analyze the expression of key molecules of cell cycle regulation. Cell lysate and protein estimation were done as published [16]. Protein lysates (60 μg) were resolved in 12% denaturing SDS PAGE gels and transferred onto nitrocellulose membrane followed by blocking with 5% non-fat milk powder. Further, it was incubated with appropriate primary and secondary antibodies and visualized by ECL detection system.

### Combination index analysis

To evaluate the nature of interaction between fisetin-doxorubicin and acacetin-doxorubicin, multiple drug effects analysis of Chou and Talalay were used. The value of combination index (CI)<1, CI = 1 and CI>1 indicates synergistic, additive and antagonistic interactions, respectively [17]. The CI value were calculated by using CampuSyn software using viable cells from trypan blue assay.

### Clonogenic assay

A total 600 cells of A549 and H1299 were seeded onto 6-well culture plate and analyzed for their clonogenic potential following the method published by Singh et al [18]. After every three days, media were changed with respective treatments. Colonies containing 50–200 cells were counted under inverted phase contrast microscope at 100× magnifications. Entire plate surface area was scored for colonies and each treatment was done in triplicate and repeated twice [18].

### Measurement of doxorubicin efflux

Briefly 3×10^5^ cells were incubated with doxorubicin for 3 h and 15 mins before harvesting the cells, and maxiumum of 0.1% DMSO was present in all the treatment groups. Further cells were washed in 1× PBS and treated with 25 μM acacetin and 25 μM silibinin in PBS and kept at 37°C under shaking condition at 50 rpm in dark. Media were collected after 5 mins, 30 mins and 120 mins and centrifuged at 6800g. Doxorubicin fluorescence was measured by spectrofluorophotometer (Shimandzu, RF-5301 PC) with ex = 488 ± 5 nm/em = 597 ± 5 nm. The equal volume of PBS was removed and replenished after each time point collection [19]. All the experiments were done in triplicate and repeated thrice.

### Doxorubicin influx measurement

Cells were treated with DMSO, 25 μM acacetin and 25 μM silibinin for 6 h with post treatment of 10 μM doxorubicin for 5 mins, 30 mins, 2 h, 6 h and 24 h. After each time point, cells were immediately collected, centrifuged and resuspended in PBS. Doxorubicin uptake inside the cells were examined using FACS (BD FACS Calibur^™^ from BD Biosciences using BD CellQuest^™^) with BD CellQuest^™^ Pro software (San Jose, CA, USA) in FL2-H filter [20].

### Intracellular accumulation of doxorubicin by confocal microscopy

For doxorubicin accumulation minimum 5×10^4^ cells/well in 6-well plate were seeded on coverslip and treated with 25 μM acacetin and 25 μM silibinin. After 6 h incubation, cells were washed and incubated with 10 μM doxorubicin for 2 h. Afterward, cells were washed and fixed with fixative solution (chilled methanol: acetonitrile; 1:1 v/v) for 15 mins at 4°C. Cell permeability was increased with 10 mins incubation using 0.5% Triton-X-100 at 4°C. After PBS wash, cells were stained with 300 ng/ml, 4′,6-diamidino-2-phenylindole (DAPI) solution for 30 mins at 37°C. Coverslips were mount with 70% glycerol and proceeded for confocal microscopy. Doxorubicin fluorescence was measured under confocal microscope (Nikon Eclipse TiE) equipped with a filter system (excitation filter DAPI range: 405 nm, emission filter FITC range). Images were obtained with camera via ANDOR Technology.

### Reverse transcription polymerase chain reaction (RT-PCR)

RNA isolation and RT-PCR was done using method described by Shin et al [21]. Primers used for PCR amplification of cDNA were MDR1: forward, 5’-AGGAGGCCAACATACATGCC-3’, reverse, 5’-AGAGTTCACTGGCGCTTTGT-3’ and GAPDH: forward, 5’-GGTCGGAGTCAACGGATTTGGTCG-3’, reverse, 5’-CCTCCGACGCCTGCTTCACCAC-3’.

### Statistical analysis

The data analysis were done with SigmaStat 2.03 software (Jandel Scientific, San Jose, CA). Student’s *t*-test was used to compare the statistical significance of control and treatment groups. The P-value < 0.05 was considered as statistically significant.

## Results

### Acacetin, fisetin and doxorubicin inhibited cell viability in NSCLC cells

**Fig 1:**
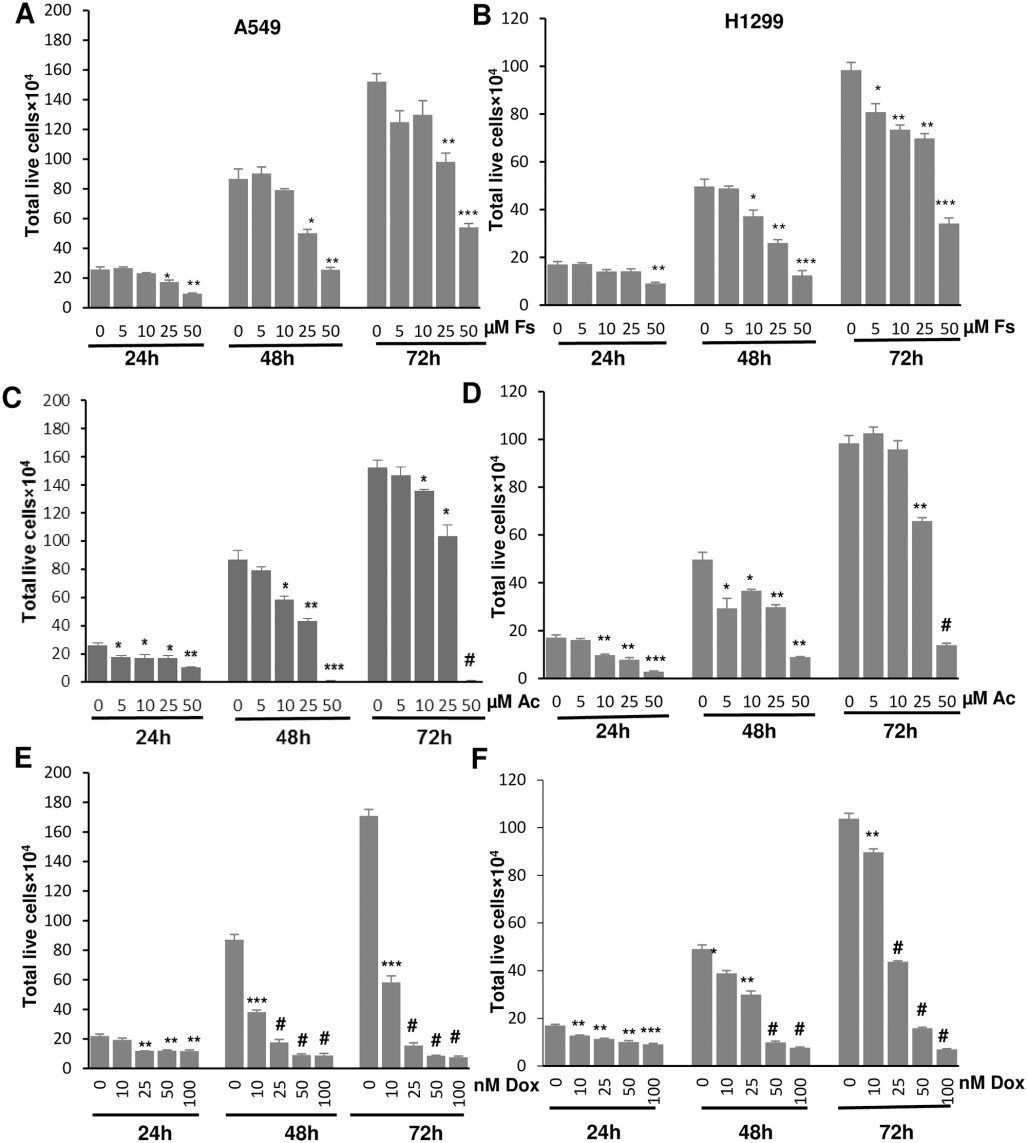
Effect of fisetin, acacetin and doxorubicin on cell growth of NSCLC cells. Total live cell number of A549 and H1299 cells treated with different concentrations of Fs (A, B), Ac (C, D) and Dox (E, F) were counted at 24, 48 and 72 h using hemocytometer by trypan blue assay. The data are presented as means of triplicate samples for each treatment. Fs, fisetin; Ac, acacetin; Dox, doxorubicin. Bars, SE; P<0.0001 (#), P<0.001 (***), P<0.01 (**), P< 0.05 (*).

To investigate the non-toxic concentrations of fisetin, acacetin and doxorubicin on A549 and H1299 cells, screening experiments were performed and suitable time points for the treatments were optimized. The concentrations ranging from 5 to 50 μM of fisetin and acacetin were used for both cell lines, whereas doxorubicin was screened with 10 to 100 nM concentrations for 24, 48 and 72 h. Fisetin, acacetin and doxorubicin treatments resulted in a time- and dose-dependent inhibition in cell growth and proliferation of A549 and H1299 cells (Fig 1). At 72 h, fisetin significantly decreased A549 cells growth by 35% (P<0.01) and 65% (P<0.001) at 25 and 50 μM concentrations, respectively (Fig 1A), whereas acacetin caused 32% (P<0.05) and 99% (P<0.001) decrease at same concentrations, respectively (Fig 1C). In H1299 cells, fisetin caused 29% and 65% decrease in cell growth (Fig 1B), but acacetin caused 33% and 86% decrease at 25 and 50 μM concentrations respectively (Fig 1D). Doxorubicin exerted significant inhibition of cell growth in both the cell lines at all the concentrations. Percentage decrease in viable cells were 66–96% in A549 cells and 14–93% in H1299 cells at 10–100 nM at 72 h (Fig 1E and 1F). These results demonstrated that IC50 values of acacetin were 28.31 μM and 31.24 μM at 72 h in A549 and H1299 cells, respectively, whereas, fisetin needed 37.50 μM and 37.78 μM of concentrations to inhibit 50% population of A549 and H1299 cells, respectively at same time point (Table 1). These results supported the earlier study showing more sensitivity of A549 cells to chemotherapeutic agents than H1299 cells [22]. In A549 cells, IC50 values of doxorubicin for 24, 48 and 72 h were 86.34 nM, 17.83 nM and 8.64 nM, respectively, whereas these were 93.86 nM, 43.28 nM and 37.12 nM for H1299 cells, respectively. Based on the cell sensitivity, we selected 10 nM of doxorubicin for A549 cells and 25 nM for H1299 cells for further experiments.

**Table 1:** Effects of fisetin, acacetin and doxorubicin on cytotoxicity IC50 values of A549 and H1299 cells.

Cell lines	Drugs	24 h	48 h	72 h
**A549**	**Fs (μM)**	38.28	33.70	37.50
	**Ac (μM)**	37.49	24.09	28.31
	**Dox (nM)**	86.34	17.83	8.64
**H1299**	**Fs (μM)**	54.54	31.10	37.78
	**Ac (μM)**	25.89	27.17	31.24
	**Dox (nM)**	93.86	43.28	37.12

Fisetin (Fs), acacetin (Ac) and doxorubicin (Dox)

### Combination of doxorubicin with acacetin synergistically inhibited the survival of cultured NSCLC cells

**Fig 2:**
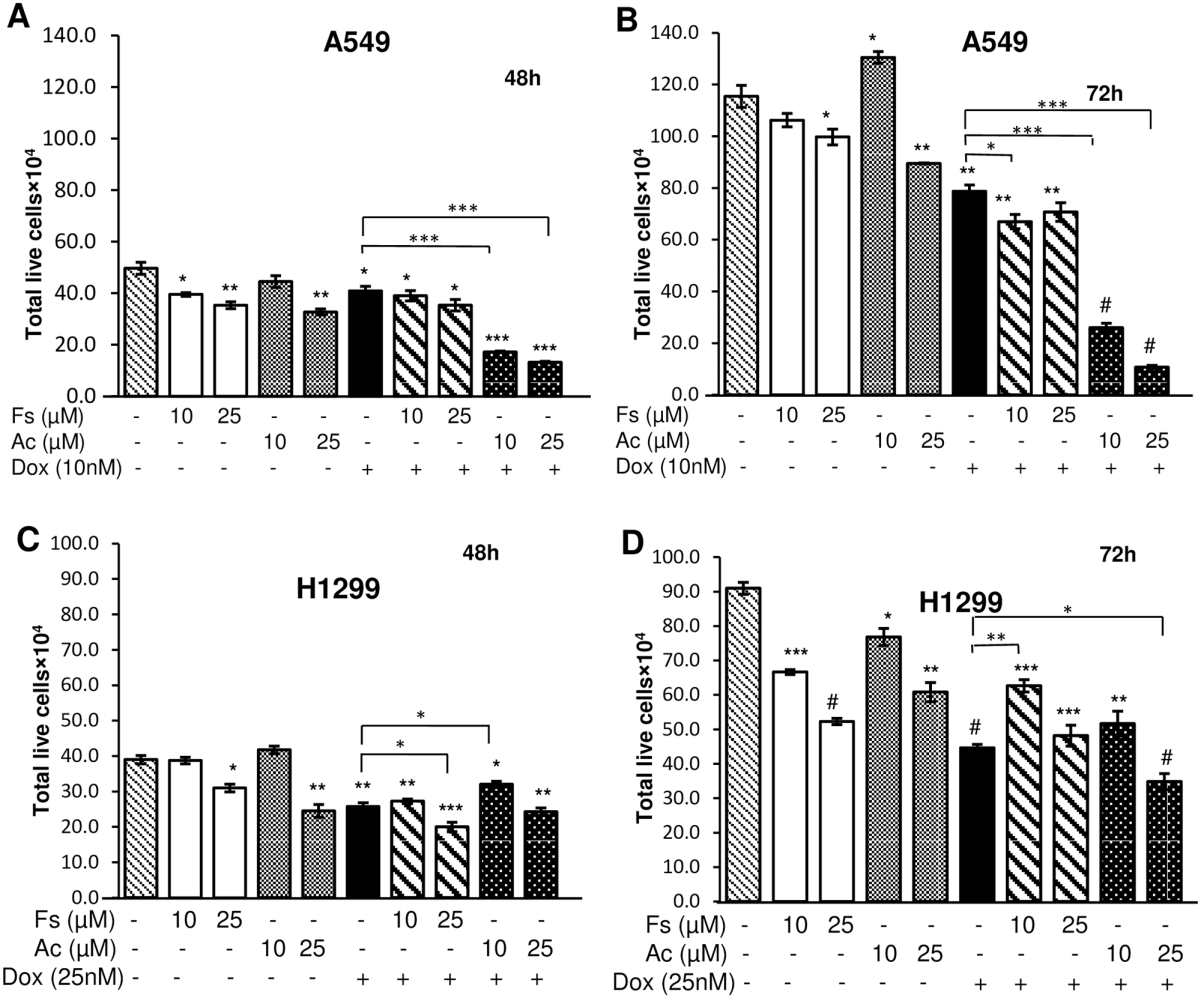
Combinatorial effects of fisetin or acacetin with doxorubicin on cell viability in NSCLC cells. A549 (A, B) and H1299 cells (C, D) were treated with 10 and 25 μM of Fs and Ac in combination with Dox (10 nM for A549 and 25 nM for H1299 cells) for 48 and 72 h. At the end of treatments, cells were processed for trypan blue assay. The data are presented as means of triplicate samples for each treatment. Fs, fisetin; Ac, acacetin; Dox, doxorubicin. Bars, SE; P<0.0001 (#), P<0.001 (***), P<0.01 (**), P< 0.05 (*).

Doxorubicin in known for its anticancer effect on various cancers, therefore enhancement of its therapeutic potential at lower doses is desired. Based on inhibitory effects, non-toxic concentrations of fisetin, acacetin and doxorubicin were selected and combined to evaluate their synergy by combination index (CI). In A549 cells, 25 μM acacetin combination with 10 nM doxorubicin caused 68% and 86% decrease of cell viability of A549 cells at 48 and 72 h, respectively as compared to 10 nM doxorubicin alone. With same concentration, fisetin-doxorubicin exerted only 13% and 10% decrease with respective time points (Fig 2A and 2B). In relatively resistant H1299 cells, 25 nM doxorubicin with 25 μM acacetin caused 6% and 22% decrease in cell viability than doxorubicin alone at 48 h and 72 h, respectively, but doxorubicin individual treatment was working more efficiently than fisetin-doxorubicin co-treatment (Fig 2C and 2D). Synergistic, additive and antagonistic effect of drug combinations was calculated with CI<1, CI = 1 and CI>1, respectively [23]. It was noted that CI value of acacetin-doxorubicin was 0.36 and 0.58 for 72 h in A549 and H1299 cells, respectively, whereas, fisetin-doxorubicin combination with same time point showed 1.62 and 0.68 CI for A549 and H1299 cells, respectively (Table 2).

**Table 2:** Combination index (CI) of the combination treatment of fisetin-doxorubicin and acacetin-doxorubicin in NSCLC cells.

**A549 cells**			**Fa = 1-fraction of surviving cells**			**Combination index (CI)**		
			**Fs (μM)**			**Fs (μM)**		
			**0**	**10**	**25**	**0**	**10**	**25**
**48h**	**Dox (nM)**	**0**	-	0.19	0.40	-	-	-
		**10**	0.26	0.30	0.47	-	1.43	1.34
**72h**	**Dox (nM)**	**0**	-	0.18	0.45	-	-	-
		**10**	0.47	0.37	0.54	-	1.67	1.62
**H1299 cells**								
**48h**	**Dox (nM)**	**0**		0.01	0.30	-	-	-
		**25**	0.22	0.24	0.42	-	1.41	1.73
**72h**	**Dox (nM)**	**0**	-	0.09	0.27	-	-	-
		**25**	0.38	0.45	0.57	-	0.82	0.68
**A549 cells**			**Fa = 1-fraction of surviving cells**			**Combination index (CI)**		
			**Ac (μM)**			**Ac (μM)**		
			**0**	**10**	**25**	**0**	**10**	**25**
**48h**	**Dox (nM)**	**0**	-	0.15	0.32	-	-	-
		**10**	0.26	0.59	0.70	-	0.56	0.53
**72h**	**Dox (nM)**	**0**	-	0.18	0.31	-	-	-
		**10**	0.47	0.72	0.90	-	0.66	0.36
**H1299 cells**								
**48h**	**Dox (nM)**	**0**	-	0.01	0.12	-	-	-
		**25**	0.22	0.32	0.42	-	1.12	1.32
**72h**	**Dox (nM)**	**0**	-	0.18	0.38	-	-	-
		**25**	0.38	0.48	0.62	-	0.72	0.58

Fisetin (Fs), acacetin (Ac) and doxorubicin (Dox)

### Doxorubicin treatment with acacetin induced cell cycle arrest and modulated the cell cycle regulators in lung cancer cells

**Fig 3:**
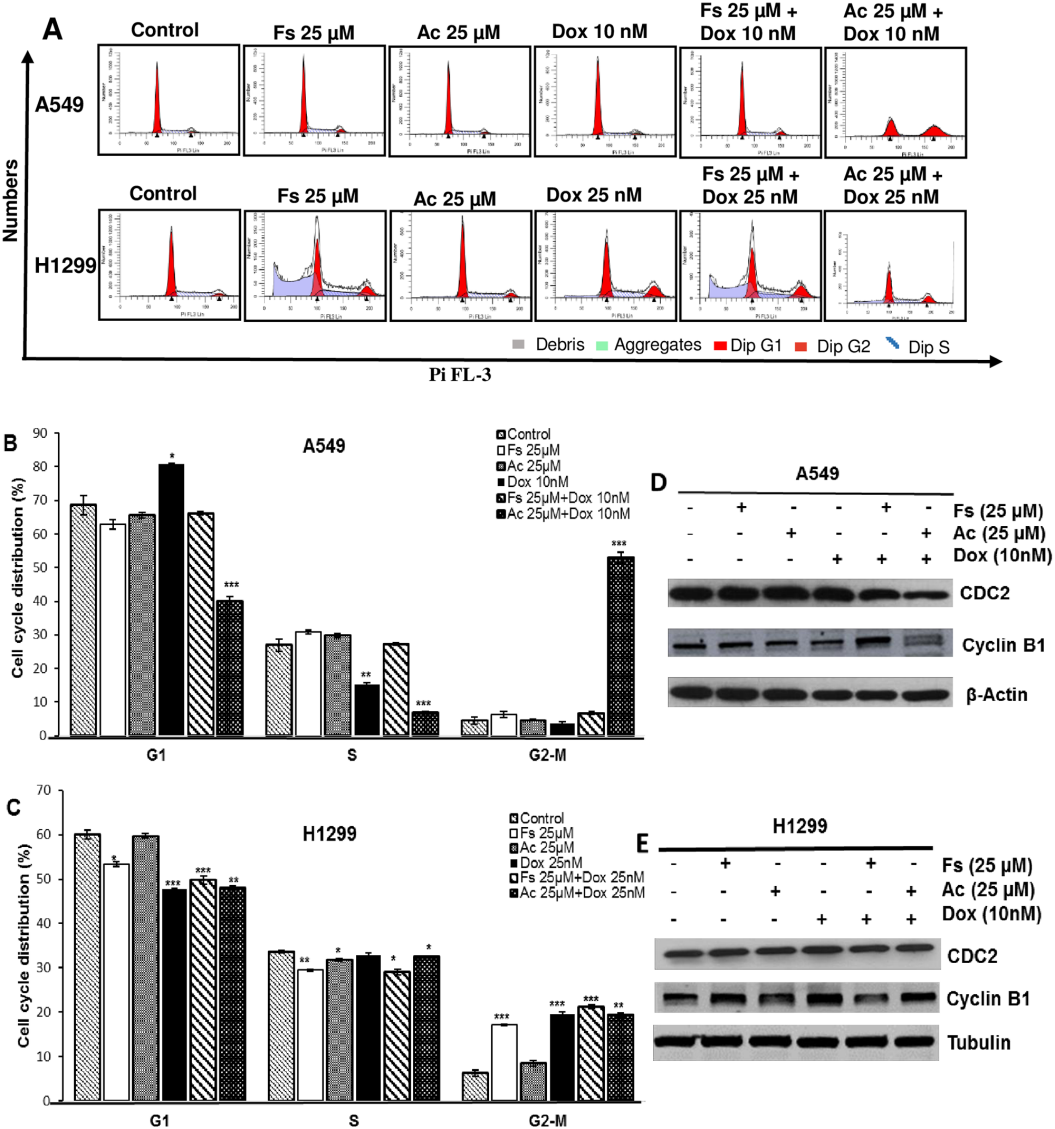
Effect of fisetin and acacetin in combination with doxorubicin on A549 and H1299 cell cycle progression. A549 (A, B) and H1299 (A, C) cells were treated for 72 h as indicated in the figure and analyzed by flow cytometry. Immunoblot analysis of cell cycle regulatory molecules involved in G2/M phase were done after 72 h of treatments in A549 (D) and H1299 cells (E). Tubulin and β-actin were used as loading control. The data is presented as means of triplicate samples for each treatment. Fs, fisetin; Ac, acacetin; Dox, doxorubicin. P<0.001 (***), P<0.01 (**), P< 0.05 (*).

Anticancer agents can elicit inhibition in tumor growth by causing arrest in particular phase of cell cycle [24,25]. Selected combinations of doxorubicin with fisetin and acacetin were used to analyze their cell cycle kinetics at 72 h. Doxorubicin at 10 nM concentration in A549 cells showed 81% G1 phase cell population (P<0.05) as compared to 69% in control, when combined with 25 μM acacetin, it induced a strong G2/M phase cell cycle arrest (53%, P< 0.001) compared to control (5%) (Fig 3B). In resistant H1299 cells, 25 μM fisetin, 25 μM acacetin, 25 nM doxorubicin and combination of doxorubicin with fisetin and acacetin showed 17%, 8%, 20%, 21% and 19% G2/M phase cell population, respectively as compared to 6% in control (Fig 3C).

Further, we analyzed the expression of cyclins and cyclin-dependent kinase (CDK) involved in G2/M phase arrest induced by acacetin-doxorubicin combination. We observed a significant decrease in the expression of CDC2 in A549 cells treated with acacetin-doxorubicin combination, whereas, acacetin and doxorubicin alone were not able to cause any change in its expression (Fig 3D). No effect was observed in the expression of Cyclin B1 and CDC2 in H1299 cells (Fig 3E). These observations suggested that G2/M arrest in A549 cells by acacetin-doxorubicin combination was mediated through modulation of CDK-cyclin complex.

### Acacetin-doxorubicin combination represses clonogenic potential of NSCLC cells

**Fig 4:**
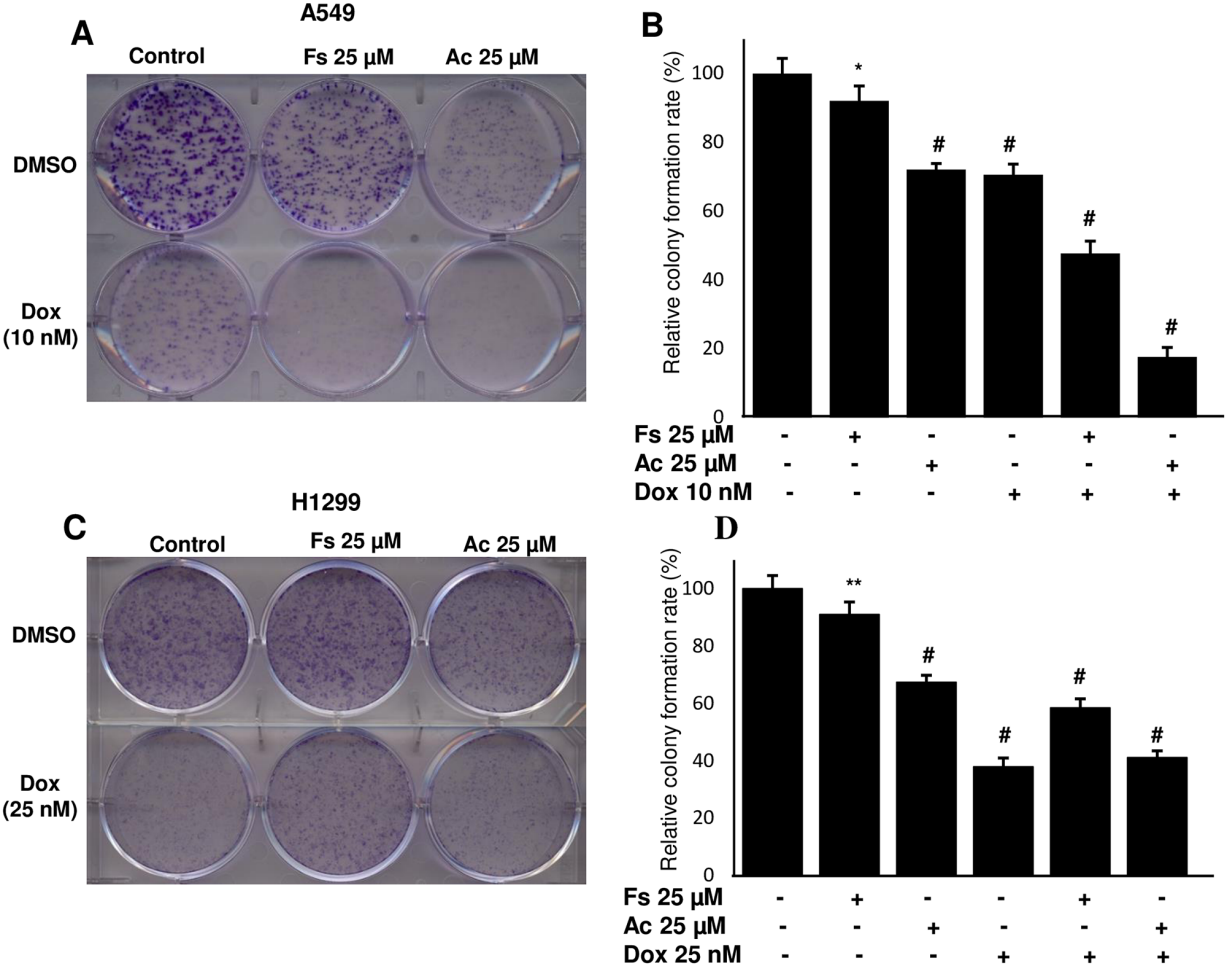
Effect of combination of fisetin and acacetin with doxorubicin on the clonogenic potential of A549 and H1299 cells. A549 (A, B) and H1299 cells (C, D) were treated with indicated concentrations of Fs, Ac and Dox in regular growth medium for 8 days. After that cells were processed/stained with crystal violet dye. Colonies size between 50–200 cells were counted and plotted (C, D). The data is presented as means of triplicate samples for each treatment. Fs, fisetin; Ac, acacetin; Dox, doxorubicin. P<0.0001 (#), P<0.01 (**), P< 0.05 (*).

Colony formation potentials of A549 and H1299 cells were studied after treatment with doxorubicin along with fisetin and acacetin. We observed that as compared to control acacetin and doxorubicin inhibited colony forming ability of A549 by 28% and 29% respectively, but acacetin-doxorubicin combination exerted 82% decrease (P<0.0001). Fisetin-doxorubicin also significantly reduced 52% colonogenicity of A549 cells (Fig 4A and 4B). In H1299 cells, acacetin-doxorubicin inhibited colony formation by 59% compared to control but it alone suppressed colonogenic potential by 62%, which indicated resistant behavior of H1299 cells toward this combination (Fig 4C and 4D).

### Acacetin-doxorubicin combination reduces doxorubicin efflux from the cells

**Fig 5:**
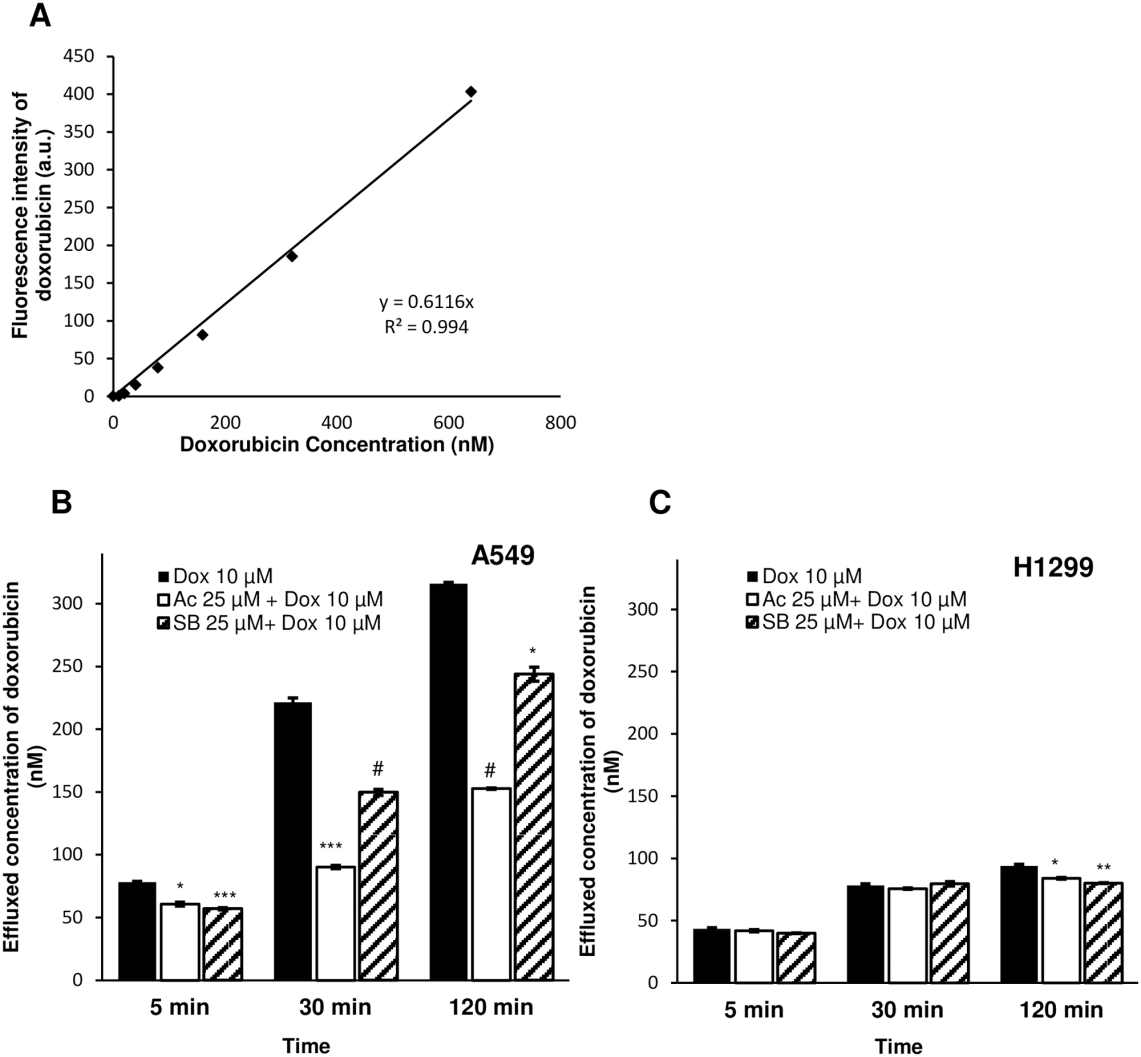
Doxorubicin efflux measurement in the cytoplasm of A549 and H1299 cells. (A) Standard curve of Dox was prepared in PBS to measure its fluorescence by spectrofluorophotometer. (B) A549 and (C) H1299 cells were pre-treated with 10 μM Dox for 2 h and washed with PBS to remove extracellular Dox. Thereafter, cells were incubated with 25 μM Ac and 25 μM SB at 37°C and 50 rpm. Cell supernatant was processed to measure effluxed Dox at different time points. Dox ex = 488 nm and em = 597 nm. Ac, acacetin; SB, silibinin; Dox, doxorubicin. The data shown are means of three independent plates. P<0.0001 (#), P<0.001 (***), P<0.01 (**), P< 0.05 (*).

There are various efflux pumps on cell membrane which play significant role to reduce intracellular accumulation of chemotherapeutic agents [26]. Resistant cells have more expression of these drug transporters compared to sensitive cells. Our results indicated that acacetin in combination with doxorubicin can enhance its cytotoxicity in NSCLC cells. To understand the possible mechanism behind the synergistic effect of doxorubicin in combination with acacetin, we examined the effect of combination on drug efflux outside the cells at different time points. Doxorubicin efflux in media was decreased by 22% (P<0.005), 59% (P<0.0005) and 52% (P<0.0001) at 5 mins, 30 mins and 120 mins, respectively in combination of acacetin-doxorubicin compared to control (Fig 5C). With increase in time, retention of doxorubicin with acacetin inside A549 cells was higher compared to silibinin treatment, which is known to inhibit drug efflux by modulating drug transporters [27]. There was not much difference observed in doxorubicin efflux in H1299 cells (Fig 5C).

### Acacetin treatment enhances doxorubicin uptake in NSCLC cells

**Fig 6:**
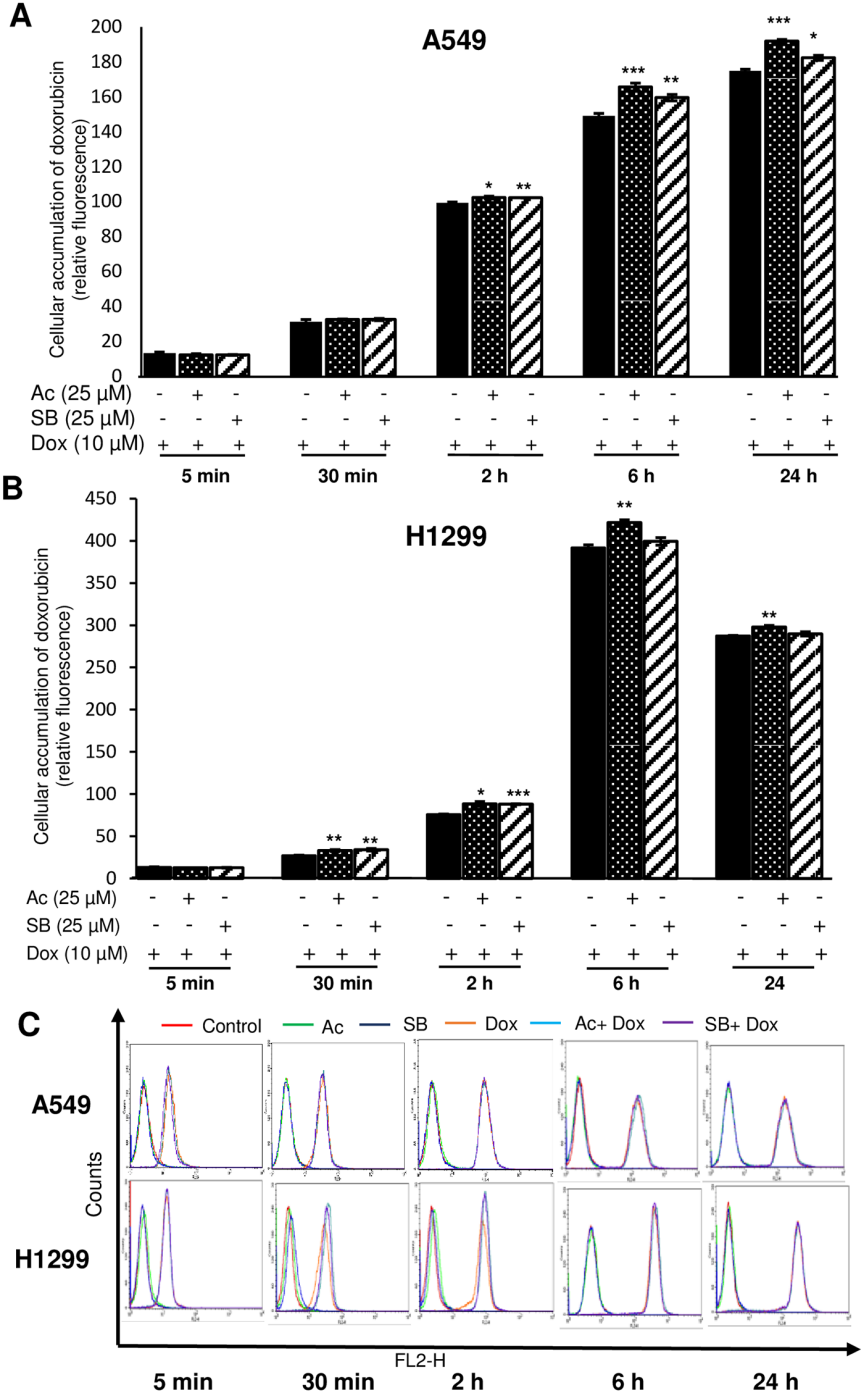
Flow cytometry analysis of doxorubicin uptake in A549 and H1299 cells. (A) A549 and (B) H1299 cells were pre-treated with 25 μM Ac and 25 μM SB for 6 h and post-incubated with 10 μM Dox. The cells were then collected at different time points and proceed for FACS analysis. (C) Representative figure of flow cytometry histogram of fluorescence intensity after respective treatments. The data is presented as means of triplicate samples for each treatment. Groups at different time points have been compared with respective Dox alone treatment. Ac, acacetin; SB, silibinin; Dox, doxorubicin. P<0.001 (***), P<0.01 (**), P< 0.05 (*).

To determine the intracellular accumulation of doxorubicin, flow cytometry and confocal cell imaging were done. As the time increased, doxorubicin accumulation inside the cells was increased significantly in both the cell lines in combination treatment (Fig 6A and 6B). Uptake of doxorubicin was significantly increased in combination of acacetin or silibinin (P<0.001 and P<0.05 for acacetin-doxorubicin and silibinin-doxorubicin, respectively) even after 24 h in A549 cells (Fig 6A), whereas, low retention of doxorubicin was observed in H1299 cells after 6 h (Fig 6B).

**Fig 7:**
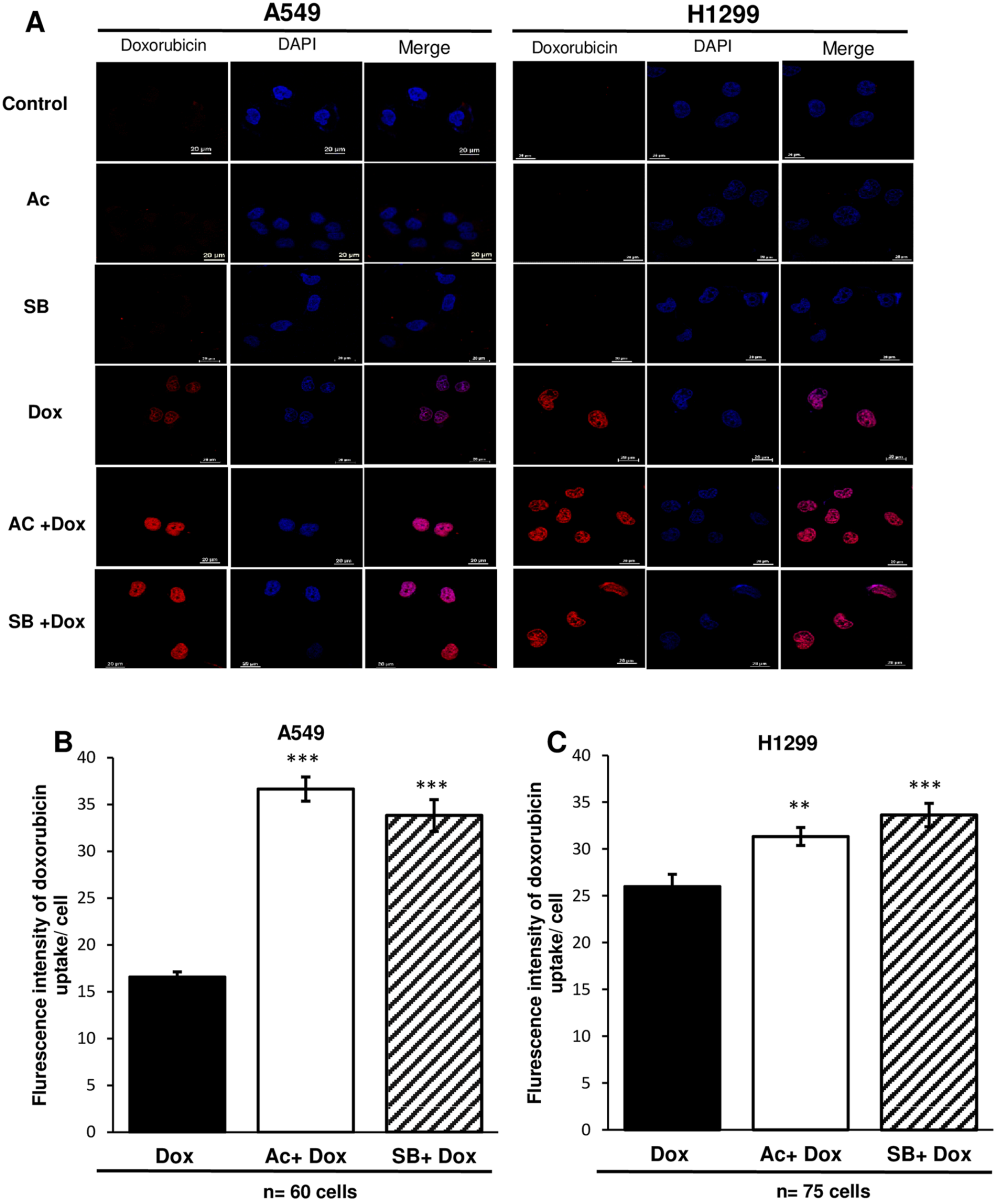
Doxorubicin uptake in A549 and H1299 cells by confocal microscopy. A549 and H1299 cells (A) cells were treated with 25 μM Ac and 25 μM SB for 6 h with 2 h posttreatment of 10 μM Dox. Afterward, cells were stained with DAPI followed by confocal imaging. Quantitative analysis of Dox fluorescence intensity in A549 (B) and H1299 (C). Treatment groups have been compared with Dox alone exposure. Ac, acacetin; SB, silibinin; Dox, doxorubicin. The quantitative data shown are means of two samples for each treatment. P<0.001(***), P<0.01(**).

In order to further investigate accumulation of doxorubicin in cytoplasm, confocal microscopy was used. In A549, more than two times increased accumulation of doxorubicin was observed in combination with acacetin, which was 8% higher than silibinin-doxorubicin (Fig 7A and 7B). In H1299, only 21% increase in uptake was observed by acacetin-doxorubicin compared to doxorubicin alone treatment (Fig 7A and 7C). Doxorubicin treatment with acacetin increased more retention of the drug in A549 as compared to H1299 cells and also in comparison to alone doxorubicin treatment.

### Combination of acacetin-doxorubicin modulate the expression of MDR1 transporter

**Fig 8:**
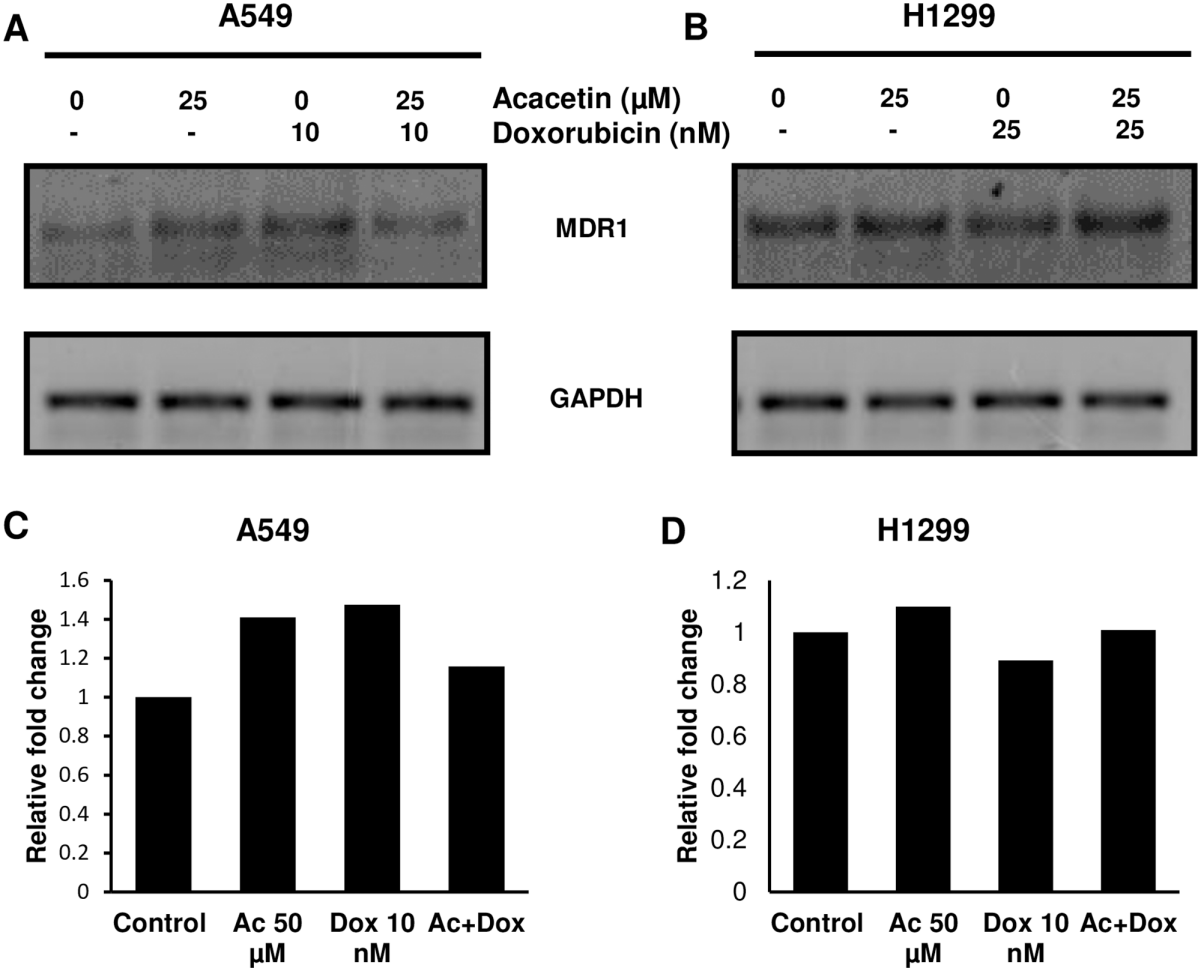
Expression of MDR1 gene by RT-PCR. A549 and H1299 cells were treated with respective treatments for 72 h and processed by RNA isolation. Semi quantitative PCR reaction of amplified MDR1 product were analysed by 1% agarose gel electrophoresis. (A) and (B) are representing the RNA expression of MDR1 gene in A549 and H1299 cells, respectively. (C) and (D) are bar diagram of densitometric value of bands representing the relative fold change in RNA expression. GAPDH was used as loading control.

Influx and efflux of any chemotherapeutic compound is controlled by various drug transporters on cell membrane. MDR1, a member of the ATP-binding cassette transporter superfamily, regulates the uptake of doxorubicin [28]. By observing significant impact of acacetin-doxorubicin combination on doxorubicin efflux and uptake, we further explored the effect of the combination on expression level of MDR1 gene at 72 h of treatment. In A549 cells, combination of acacetin-doxorubicin decreased the mRNA expression of MDR1 by 21% as compared to doxorubicin alone exposure (Fig 8).

## Discussion

Inspite of all efforts, lung cancer is still one of the most lethal cancer worldwide. It is generally diagnosed in relatively advanced stage due to lack of screening methods, where even combined chemotherapy and radiation do not work efficiently for the desired outcome. Inherent and acquired resistance further cause treatment failure in patients.

Doxorubicin is a naturally occurring anthracycline antibiotic, produced by a variant of *Streptomyeces peucetius (var*.*caesius)* and most extensively used as anticancer drug for broad spectrum of tumors including lung cancer. It is highly effective towards SCLC but represents relatively poor sensitivity towards NSCLC patients, which accounts for 4/5 of all lung cancer patients [29]. Another issue with doxorubicin use is cardiotoxicity, which is potentiated when dose of drug is increased [7]. Therefore, there is need of novel therapeutic strategy, which can reduce cytotoxicity of doxorubicin and enhance its therapeutic efficacy towards NSCLC cells. However, there are reports which concluded that the use of doxorubicin with phytochemicals such as curcumin, genistein, green tea polyphenol, quercetin, emodin and resveratrol enhance the chemosensitizing, chemopreventive and chemotherapeutic profile of doxorubicin [30]. In current study, two flavonoids, fisetin and acacetin have been investigated for their potential as chemosensitizer for doxorubicin in NSCLC cells. Two cell lines A549 and H1299 were selected for the study based on their sensitivity towards various chemotherapeutic agents. A549 cells depict sensitivity phenomena towards many compounds including cisplatin, whereas H1299 is relatively resistant for them [22]. The value of IC50 of doxorubicin at 72 h was 23% more in H1299 compared to A549 cells. From the results, it is demonstrated that acacetin with doxorubicin at non-toxic concentration, potentiated its growth inhibitory effect on A549 cells more efficiently than H1299 cells. Doxorubicin with acacetin showed best synergism (CI<1) in both the cell lines at 48 h and 72 h, whereas, its combination with fisetin exerted antagonistic effect (CI>1) except for 72 h in H1299 cells.

Inhibition of tumor growth can cause arrest in cell cycle progression. Therefore, we analyzed the effect of doxorubicin with small molecules on cell cycle kinetics. Significant inhibition of cell viability in A549 cells with acacetin-doxorubicin combination, was supported by G2/M arrest in cell cycle, which was not observed in H1299 cells. Transition from one phase to another phase of cell cycle is regulated by cyclin and cyclin-dependent kinases (cdks). Cyclin B1-dependent CDC2 kinase activation control the progression of cells from G2 to M phase. There was a decrease in expression of CDC2 in acacetin-doxorubicin combination in A549, although CDC2 activity is regulated by its binding to cyclin B1, therefore, downregulation of CDC2 might cause G2/M arrest. This complex remained unchanged in H1299 cells. Tumor growth and progression is also dependent on colony forming ability of cancer cells. Co-treatment of acacetin or fisetin with doxorubicin decreased the colony forming ability of NSCLC cells, wherein acacetin showed better effect than fisetin.

Cancer cells have more expression of drug transporters, which effectively transport the therapeutic compound outside the cells and help cancer cells to confer resistant towards them. These active transporters decrease the intracellular accumulation of drugs including anthracyclines, cisplatin, taxanes, vinca alkaloids, methotrexate and reduce the induced cytotoxicity [31]. This study examined the effect of small molecule on the phenotype of drug resistance in NSCLC cells into two ways. Firstly, by measuring the effect of acacetin on doxorubicin efflux mediated by drug transporters and secondly, by observing the doxorubicin accumulation inside the cells. Results showed that pre-incubation of NSCLC cells with doxorubicin for 3 h could sufficiently accumulate drug inside the cells and only 30 mins exposure of these cells to acacetin could significantly reduce efflux of doxorubicin in A549 cells. The modulatory effects of acacetin on transporters were further confirmed by increase in fluorescence of doxorubicin inside NSCLC cells.

This decreased efflux and increased uptake of doxorubicin by acacetin is regulated by MDR1 trasporter in NSCLC cells. Downregulation of MDR1 gene at mRNA level by acacetin-doxorubicin combination increased the retention of doxorubicin inside the cells, which may further increase the efficacy of doxorubicin by causing more cytotoxicity in NSCLC cells.

Overall, these findings suggest that acacetin could be used as promising combination agent for doxorubicin to enhance the therapeutic potential of doxorubicin at lower doses in NSCLC cells. At molecular level, it is revealed that combination of these two drugs promote the effect of each other synergistically. Their combination can strongly inhibit cell proliferation and cause cell cycle arrest. There increased potential is mediated at molecular level by modulating the activities of drug transports These findings warrant pre-clinical efficacy study of the combination using relevant *in vivo* mouse model for the clinical relevance of the observations. This study may help to promote the use of acacetin in combination with doxorubicin to increase the anticancer efficacy of doxorubicin at lower doses with reduced side effects.

### Conflict of interest

All the authors confirm that there are no known conflicts of interest associated with this publication.

## Supporting information

S1 File Supporting information. Minimal data set for Figs 1–8.
(XLSX)

## Abbreviations

MDR: Multidrug resistance
NSCLC: non-small-cell lung carcinoma
SCLC: small-cell lung carcinoma
